# The Relevance of miRNA-21 in HSV-Induced Inflammation in a Mouse Model

**DOI:** 10.3390/ijms16047413

**Published:** 2015-04-02

**Authors:** Bunsoon Choi, Hyoun-Ah Kim, Chang-Hee Suh, Hae Ok Byun, Ju-Yang Jung, Seonghyang Sohn

**Affiliations:** 1 Department of Microbiology, Ajou University School of Medicine, Suwon 443-380, Korea; E-Mails: blueppang@ajou.ac.kr (B.C.); 97360522@hanmail.net (H.O.B.); 2 Department of Rheumatology, Ajou University School of Medicine, Suwon 443-380, Korea; E-Mails: nakhada@ajou.ac.kr (H.-A.K.); chsuh@ajou.ac.kr (C.-H.S.); serinne20@hanmail.net (J.-Y.J.); 3 Department of Biomedical Sciences, Graduate School of Ajou University; Suwon 443-380, Korea

**Keywords:** microRNA-21, herpes simplex virus-induced inflammation, mouse model, Behçet’s Disease, miR-21 antagomir

## Abstract

The purpose of this study was to clarify the correlation between microRNA-21 (miR-21) expression and inflammation in a herpes simplex virus (HSV)-induced Behçet’s Disease (BD) mouse model. miR-21 was compared between BD patients and healthy controls in peripheral blood mononuclear cells (PBMC). For miR-21 inhibition, miR-21 antagomir was applied to BD mice. The change of symptoms was monitored. The levels of cytokines and related molecules were determined by ELISA and real time qPCR. Treatment with colchicine or pentoxifylline down-regulated the level of miR-21 with improved symptoms in mice. miR-21 inhibition was accompanied by down-regulated serum levels of IL-17 and IL-6. The expression levels of PDCD4, RhoB, PD-1, IL-12p35, and toll-like receptor-4 were also regulated by miR-21 inhibition. miR-21 was correlated with HSV-induced BD-like inflammation in mice and BD patients. The expression of miR-21 was regulated by antagomir in mice.

## 1. Introduction

MicroRNAs (miRNAs) are small non-coding RNAs that play a crucial role in regulating a wide range of cellular processes, such as host responses to viral infection, innate immunity, and apoptosis through the inhibition of mRNA translation [1]. Recent studies suggest that miRNAs play an important role in immunity and autoimmunity. miRNAs function in shaping immunity by regulating a repertoire of genes expressed in immune cells. Expression profiling has shown distinct patterns of miRNA expression in different hematopoietic cell lineages. A single miRNA can have substantial effects in regulating immune responses. For example, miRNA-155 expression was significantly decreased in ocular Behçet’s Disease (BD), and miR-146a gene polymorphism was also associated with ocular BD [2,3].

miRNA-21 (miR-21) is one of the first microRNAs identified [4]. It is a key regulator of oncogenic processes [5,6]. It is one of the most frequently up-regulated miRNAs in solid tumors [7]. Over-expression of miR-21 in cancer cells promotes cell survival by decreasing apoptosis [8]. miR-21 has been reported in gastric [9], renal [10], esophageal [11], colon [12], lung [13], pancreas [14], tongue [15], prostate [16], breast [17], and brain [18] cancers. In the immune system, deregulation of miR-21 has recently been reported to be related to malignancies such as B cell lymphoma and Hodgkin’s lymphoma [19,20]. miR-21 is one of the most abundant miRNAs in T cells, specifically in effector T cells, indicating that miRNA profile changes dynamically during T-cell differentiation [21]. According to Svrcek *et al.* [22] miR-21 was increased in inflammatory bowel disease (IBD) with or without colorectal cancer. miR-21 was also over-expressed in the inflamed colonic mucosa of patients with ulcerative colitis (UC) [23] and colonic Crohn’s disease (CD) [24]. These reports showed that miR-21 extendeds to the non-neoplastic mucosa. miR-21 was over-expressed in atopic eczema and psoriasis compared to its expression in healthy controls [25]. In addition, miR-21 was involved in inflammatory responses during the innate immune response to aerosolized lipopolysaccharide (LPS) in mouse lung [26]. Further studies are needed to delineate the exact role of miR-21 in the chronic inflammation *in vivo*.

The etiology of BD is still unclear. Many factors have been demonstrated as relevant factors of BD [27,28,29,30]. However, the etiologic and pathologic progress of this disease remains to be elucidated. Viral infection has long been hypothesized to be one of the main factors of BD. It has been verified by the detection of herpes simplex virus (HSV) in patients with BD since it was first proposed by Hulûsi Behçet [31,32,33,34]. Furthermore, inoculation of mice with HSV resulted in the development of BD-like symptoms [35]. Therefore, HSV-induced mouse has been widely used as an animal model of BD [36]. Manifestations in mice involve multiple symptoms with chronic inflammation similar to that of human patients with BD [37]. In this study, antagomir-mediated inhibition of miR-21 was first demonstrated in a mouse model for therapeutic application. miR-21 inhibition correlated to the improvement of inflammatory BD-like symptoms through regulating cytokine expression and toll-like receptor 4 (TLR4).

## 2. Results

### 2.1. Different Expressions of miRNA Regarding BD Symptoms

**Figure 1:**
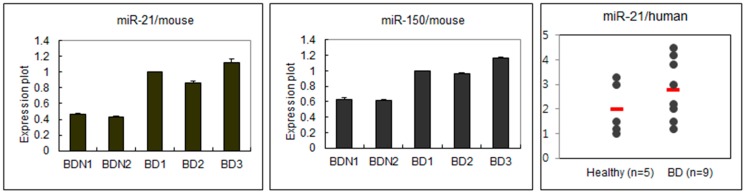
Expressions of miR-21 and miR-150 in BD mice and miR-21 in BD patients. In mice, the expression of miR-21 and miR-150 was higher in BD than BDN. In human, miR-21 was also higher in BD patients than healthy control.

In order to find the difference in miRNA expression patterns between BDN (BD Normal: HSV inoculated, but asymptomatic healthy mice) and BD mice, real time PCR was used to determine the expression levels of several miRNAs in lymph nodes. The expression of miR-21 and miR-150 were significantly (*p* < 0.05) different between BDN and BD. miR-21 and miR-150 in BD were highly expressed compared to those in BDN (Figure 1). In PBMC of human patients with BD (*n =* 9), the expression of miR-21 was also higher than that in healthy normal (*n =* 5) (*p =* 0.12).

### 2.2. miRNA Expression Was Regulated by Medication

**Figure 2:**
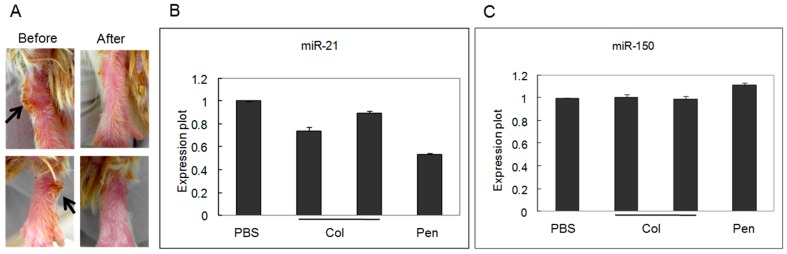
Cutaneous manifestation of mice was improved after pentoxifylline medication (arrows: skin lesion) (**A**); Expression of miR-21 was down-regulated by medication with either colchicine or pentoxifylline (**B**); miR-150 expression was not affected after medication (**C**).

To determine whether medication could regulate miRNAs expression in BD mice, mice were treated with colchicine (*n =* 6) or pentoxifylline (*n =* 5). Figure 2A show the changes of skin lesion on ankle after treatment with pentoxifylline. The expression levels of miR-21 and miR-150 were then analyzed by real-time PCR. miR-21 expression was significantly (*p* < 0.05) down-regulated after treatment with either colchicine or pentoxifylline. However, miR-150 expression was unchanged after the treatment of either of the two medications (Figure 2).

### 2.3. Correlation of miR-21 Inhibition with BD Symptoms

**Figure 3:**
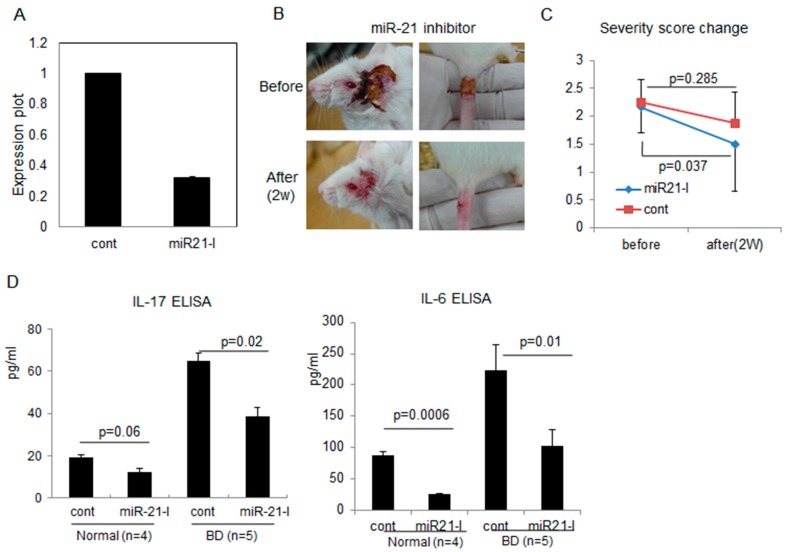
miR-21 antagomir (miR-21 inhibitor, miR21-I) inhibited miR-21 expression (**A**); improved BD-like symptoms (**B**); and decreased BD severity score (**C**) as well as serum levels of IL-17 and IL-6 (**D**).

To determine whether miR-21 inhibitions was possible in normal mice *in vivo*, miR-21 antagomir (miR-21 inhibitor, miR21-I) was injected intraperitoneally (ip) with transfection reagent three times, with 2 days intervals (days 0, 2, and 4). The lymph nodes (LN) were isolated one day after the last injection (day 5) and subjected to real time PCR analysis to detect miR-21 expression level. miR21-I injection significantly (*p* < 0.05) inhibited miR-21 expression compared to the transfection reagent injected control group in normal healthy mice (Figure 3A). In addition, miR-21 injected BD mice showed improvement of BD-like symptoms (Figure 3B). The disease severity score was decreased significantly (*p =* 0.037) from 2.18 ± 0.41 to 1.5 ± 0.55 (Figure 3C). In addition, pro-inflammatory cytokine IL-17 was significantly (*p =* 0.02) decreased to 38.48 ± 5.5 pg/mL in miR21-I injected BD mice compared to 64.67 ± 10.9 pg/mL in transfection reagent (TR) injected control BD mice (*n =* 5 in each group, Figure 3D). In normal healthy mice, miR21-I also inhibited serum IL-17 expression. IL-6 was also significantly (*p =* 0.01) down-regulated to 103.56 ± 25.44 pg/mL after miR21-I injection compared to TR injection (222.83 ± 40.62 pg/mL) in BD mice. In normal mice, serum level of IL-6 was also significantly (*p =* 0.0006) down-regulated after inhibition of miR-21 (Figure 3D).

### 2.4. Up-Regulated Genes after miR-21 Inhibition

**Figure 4:**
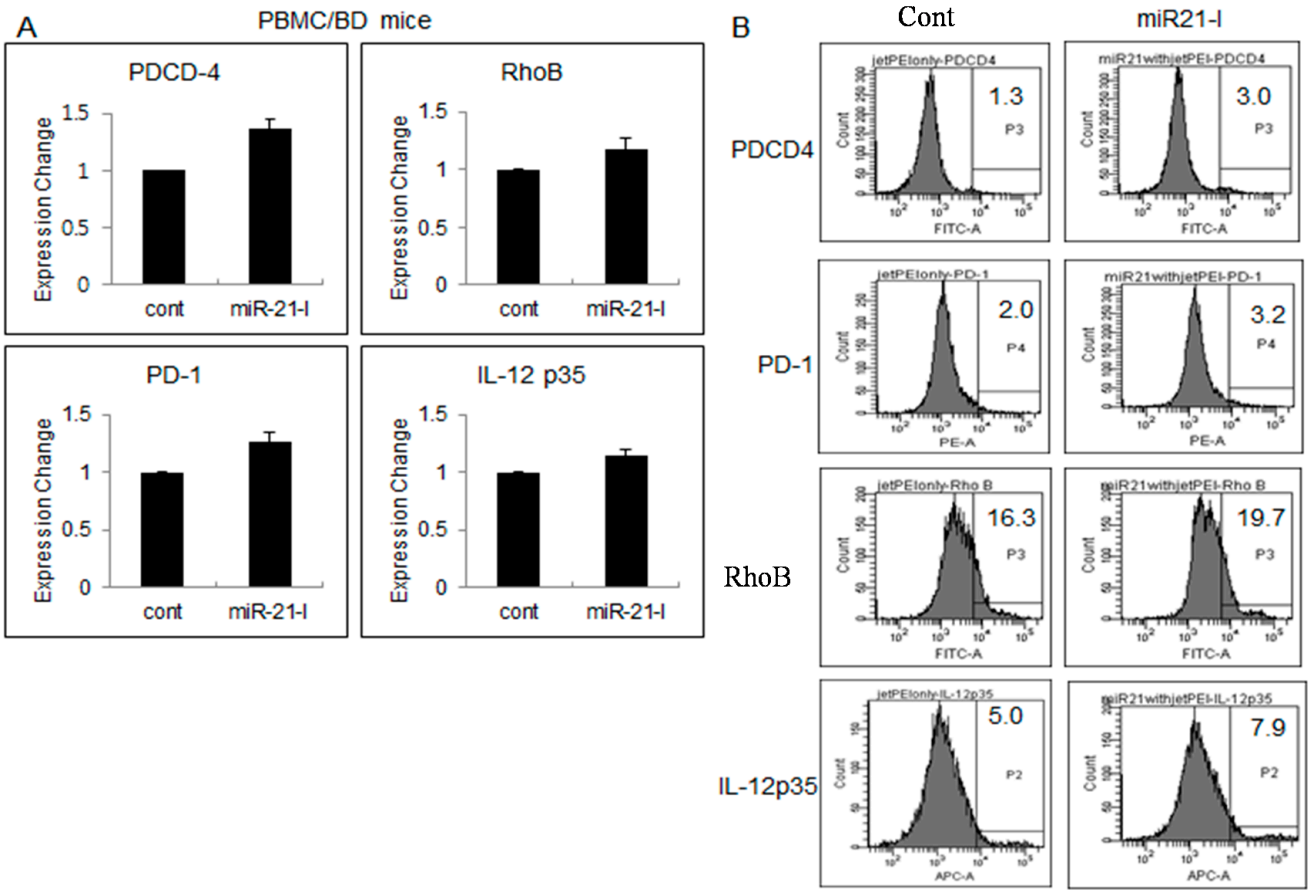
miR-21 inhibition by antagomir up-regulated several genes in BD mice by time qPCR in BD mice (**A**); Regulated genes by miR21-I was reconfirmed by protein expression with FACS analysis in PBMC isolated from BD mice (**B**).

In order to confirm the changes of target molecules after inhibition of miR-21, real time-qPCR was applied to BD mice (Figure 4A). miR21-I or TR was ip injected three times with two-day intervals. At 4 h after the last injection, the mice were sacrificed and used for analysis. Programmed cell death 4 (PDCD4) is known to be up-regulated during apoptosis [38] as a functionally important target of miR-21 [39]. In PBMC after miR21-I injection, PDCD4 expression was increased in BD mice. In addition, the expression levels of RhoB, PD-1, and IL-12p35 were also increased in BD mice. RhoB is known as a target of miR-21 [40]. The mRNA expression of RhoB is consistently up-regulated after miR21 inhibition in BD mice. According to Lu *et al.* [41] IL-12p70 was higher in dendritic cell culture conditioned media in miR-21 knockout (miR^−/−^) mice than in miR-21^+/+^ mice. Our data also showed increased IL-12p35 mRNA levels in miR-21 inhibited mice. PD-1 (programmed cell death 1) was also increased in miR-21 inhibited mice. For the confirmation of protein expression after miR-21 inhibition, flow cytometric analysis was applied in isolated PBMC of BD mice. The frequencies of PDCD4 positive cells were 3.0% ± 1.5% in miR21-I treated group compared to 1.5% ± 1.1% of TR treated control group. The frequencies of RhoB, PD-1, and IL-12p35 positive cells were also up-regulated in miR21-I group as you can see in Figure 4B.

### 2.5. miR-21 Inhibition Down-Regulated TLR4 in Normal Healthy Mice

**Figure 5:**
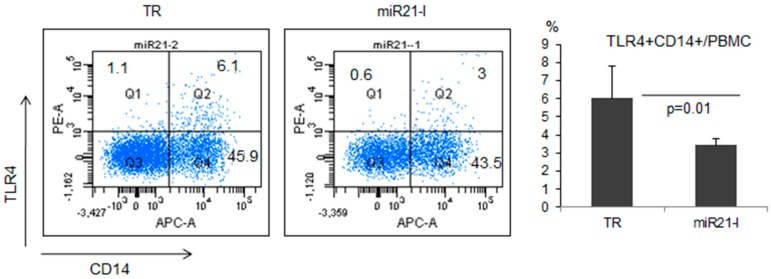
The frequencies of toll-like receptor (TLR)-4 positive cells were down-regulated after miR-21 inhibition by treatment of miR-21 antagomir in mice.

Toll-like receptors (TLR) play an essential role in the innate immune response [42]. Specifically, TLR4 was shown to be involved in inflammatory responses [43] in BD patients and mouse model [44,45]. To determine the correlation between miR-21 and TLR4, the frequencies of TLR4+ cells were analyzed among CD14+ cells in PBMC of miR21-I injected normal healthy mice (*n =* 6). Flow cytometry analysis revealed that the frequencies of TLR4+ cells were significantly (*p =* 0.01) down-regulated in miR21-I injected mice (3.44% ± 0.35%) compared to TR injected control mice (6.03% ± 1.76%, Figure 5).

## 3. Discussion

miRNA has been altered in the regulation of immune responses [46]. miRNA is associated with the development of lymphoid cells [47]. Increased expressions of miR-155, miR-181a, and miR-17~92 clusters have been shown to promote the survival and proliferation of lymphoid cells [48,49,50]. Decreased miR-155 expression was reported in ocular BD patients [2]. According to recent reports, miR-21 also contributes to the development of inflammatory bowel disease (IBD) [22] and allergic airway inflammation [51]. Our study showed that colchicine or pentoxifylline treatment ameliorated BD-like symptoms accompanied by down-regulation of miR-21 expression in PBMC of mice. The expression of miR-150 was not affected by colchicine or pentoxifylline treatment. miR-21 inhibition by intraperitoneally injection of miR-21 antagomir showed down-regulation of miR-21 in normal healthy mice. This miR-21 antagomir injection brought an improvement of BD-like symptoms accompanied by decreased serum level of IL-17 and IL-6. In the miR-21 inhibited group, mRNA expressions of PDCD4, RhoB, PD-1, and IL-12p35 were up-regulated compared to the control injected group in normal and BD mice. The frequencies of TLR4+ cells were also down-regulated after miR-21 inhibition in BD mice.

According to Lu *et al.* [41], the main function of miR-21 is to regulate the IL-12/IFNγ axis and act as a central regulator of Th1 and Th2 responses to allergens in delayed type hypersensitivity and allergic inflammation. They analyzed IL-12, TNFα, IL-6, and IL-23. In our experiment, cytokine IL-17 was lower in miR-21 antagomir treated mice. Excessive CD4^+^ T cells producing IL-17 were found in patients with BD [52]. Serum IL-17 levels were markedly up-regulated in BD [53]. Patrick *et al.* [54] showed that miR-21 antagomir might be therapeutically useful in preventing heart failure in mice.

miR-21 exhibits an anti-angiogenic function by targeting RhoB expression in endothelial cells through the repression of RhoB [55]. It has been shown that RhoB is a direct target of miR-21 in angiogenesis [55]. The expression of RhoB in PBMC of normal and BD mice was also up-regulated after miR-21 inhibition in our experiment. PD-1 deficient mice developed severe arthritis accompanied by increased miR-21 [56]. In our result, PD-1 was also negatively correlated with miR-21 expression. Merline *et al.* [57] described that the down-regulated miR-21 increased the abundance of PDCD4. Similarly, PDCD4 was up-regulated after miR-21 inhibition in the present study. Inflammation can be explained by the excessively produced inflammatory cell accumulation and delayed apoptosis of inflammatory cells. Taken together, our data support the previously reported results that miR-21 increases cell proliferation and decreases cell apoptosis [58].

Our present study showed that the level of miR-21 was correlated to HSV-induced inflammation in a BD mouse model and BD patients. There was no report related to miR-21 in BD patients or HSV-induced inflammation.

## 4. Experimental Section

### 4.1. Human Materials

Five patients with BD who presented for the first time or were monitored at the Department of Rheumatology, Ajou University Hospital were enrolled in this study. Clinical characteristics and therapeutic histories of these patients are shown in Table 1 and Table 2. According to the International Study Group for BD criteria, the presence of any two of the following symptoms in addition to recurrent oral ulcerations is considered to be sufficient for a BD diagnosis: recurrent genital ulceration, uveitis, large-vessel vasculitis, cutaneous erythema nodosum, and arthritis. These patients (*n =* 9, 8 females and 1 male, 46.2 ± 10.5 years) had at least one of the BD symptoms despite treatment. This study was approved by the Institutional Review Board. Informed consent was obtained from patients prior to enrollment into the study. The healthy control group (*n =* 5, 43.4 ± 18.4 years) consisted of 4 female and 1 male subjects.

**Table 1:** Clinical characteristics of Behçet’s Disease patients.

Patient	Age	Sex	OU	GU	Arthritis	GI	NEUR	VAS	OL	Pathergy	HLA-B51	EN	ESR	CRP
KSY	63	F	+	+	+	−	−	−	−	−		−	25	0.03
CMR	36	F	+	+	+	+	−	−	−	−		+	55	0.5
HHK	51	F	+	+	+	−	−	−	−	−		+	71	2.93
JYS	32	F	+	+	+	−	−	−	−	−	+	+	70	0.14
LJH	42	F	+	+	+	−	−	−	−	−	+	+	18	0.07
SJO	55	F	+	−	+	−	−	−	−	−	+	+	21	0.52
SKH	53	M	+	−	+	−	+	−	−	−	+	+	20	0.09
KSM	35	F	+	−	+	−	−	−	−	−	+	+	24	0.05
LMS	49	F	+	−	+	−	−	−	−	−	+	+	24	0.7

M: male; F: female; OU: oral ulcers; GU: genital ulcers; GI: gastrointestinal inflammation; NEUR: neurological involvement; VAS: vasculitis; OL: ocular lesions; EN: Erythema nodosum; ESR: Erythrocyte sedimentation rate; CRP: C-reactive protein.

**Table 2:** Therapeutic histories of Behçet’s Disease patients.

Patient	Colchicine	Steroid	Azathioprin	Bucillamine	HCQ	Minocycline	NSAIDs	SZP
KSY	+	+	+	+	+	−	+	+
CMR	+	+	+	−	−	+	−	+
HHK	+	+	−	−	−	+	+	+
JYS	+	+	+	−	−	+	+	+
LJH	+	+	−	−	+	−	+	+
SJO	+	+	−	+	+	−	+	+
SKH	+	+	−	+	−	−	+	+
KSM	+	+	−	−	+	−	+	+
LMS	+	+	−	−	−	−	+	+

HCQ: hydroxychloroquine; NSAIDs: nonsteroidal anti-inflammatory drugs; SZP: Sulphasalazine.

### 4.2. Animals

In this study, 4- to 5-week-old ICR male mice were used. Animals were handled in accordance with a protocol approved by the animal care committee of the Ajou University School of Medicine (Suwon, Korea) (Institutional Approval Number: AMC-101).

### 4.3. Clarification of BD, BDN, and Normal Mouse

The earlobes of 4 to 5 weeks’ old mice were scratched with a needle and inoculated with 1 × 10^6^ plaque-forming units (p.f.u.)/mL of HSV-1 (F strain) which had been grown in Vero cells as described previously [35]. Virus inoculation was performed twice with a 10 day interval followed by 32 weeks of observation. Multiple symptoms were observed after HSV-1 inoculation of mice. BD symptoms in human patients (mouth ulceration, genital ulceration, erythema, skin pustules, skin ulceration, joint arthritis, diarrhea, red eye, reduced vision, loss of balance, discoloration and swelling of the face) were selected. A mouse with two or more symptoms was considered as a BD mouse. HSV-1 infected but asymptomatic mice were used as BD normal (BDN). Mice without HSV-1 infection were used as control.

### 4.4. Severity Score of BD Mouse

The presence of each symptom in a BD mouse was scored as one. The sum of these scores in each mouse was used to determine the severity of BD [59]. The severity score of individual BD mice varied from two to four. The severity of BD was followed by determining the Behçet’s disease current activity form 2006 as outlined on the BD activity form prepared by the International Society for Behçet’s Disease (www.behcet.ws/behcetwsData/Uploads/files/BehcetsDiseaseActivityForm.pdf).

### 4.5. Medication of BD-Like Symptoms

BD mice were treated with PBS, colchicine (2 μg/mouse), or pentoxifylline (400 μg/mouse) for five consecutive days by oral administration. The changes in BD-like symptoms were then observed. Mice were sacrificed for further laboratory analysis.

### 4.6. miRNA Inhibition

In order to inhibit miR-21 expression, miR-21 antagomir was injected intraperitoneally three times to normal and BD mice with 2 days intervals. The injection was started from the day of BD symptom appearance. At 24 h after the final injection, the mice were sacrificed. PBMC and serum were collected for further analysis. The experimental group was composed of control, transfection reagent treatment without antagomir, and antagomir for miR-21 inhibition (miR21-I) (1 μM).

### 4.7. Reverse Transcriptase (RT)-PCR

For mRNA analysis, total RNA was isolated from lymph nodes treated with either miR21-I or transfection reagent using TRIZOL (Life Technologies, Helgerman, CT, USA) according to the manufacturer’s recommendations. The final RNA amount was spectrophotometrically determined at 260/280 nm. Reverse transcription was performed with AccuPower^®^ RT PreMix kit (USA Bioneer, Inc., Alameda, CA, USA) and oligo-dT to generate cDNA. Next, the cDNA was amplified by polymerase chain reaction (PCR) using gene-specific primers (Table 3). Amplified PCR products were then electrophoresed on 1.5% agarose gel and stained with ethidium bromide.

**Table 3:** The used primers for reverse transcription PCR (RT-PCR) and real-time quantitative PCR.

Genes	Primers
*PD-1*	(F) TCGTGGTAACAGAGAGAATCCT (R) TTCAGAGTGTCGTCCTTGCTT
*PDCD4*	(F) TTGGCAGTGTCCTTAGCCTT (R) GGCTAGCTCAGGGAGATCCT
*RhoB*	(F) CCCAGTGTCTGTGTGTGTCC (R) TGAGGCCTGGCTCTTTAGAA
*IL-12p35*	(F) TAGATGCTACCAAGGCAC (R) ATCACGCTACCTCCTCTT

### 4.8. Real-Time Quantitative PCR (qPCR)

For Real-Time Quantitative PCR, the quality of RNA was assessed as the absence of smear of 18S and 28S bands analyzed by Bioanalyzer 2100 (Agilent, Santa Clara, CA, USA). cDNAs were synthesized from 1 μg of total RNA according to the manufacture’s protocol (High Capacity RNA-to-cDNA Kit, Applied Biosystems, Waltham, MA, USA). Real-time PCR was performed in triplicates in 384-well plates. A high through-put analysis was performed by using ABI Prism 7900 Sequence Detection System (PE Applied Biosystems, Foster City, CA, USA) and white colored 384-well plates (ABgene, Hamburg, Germany) for intensification of the fluorescent signals. The fluorescence emission from each sample was collected and the quantitative data were analyzed using Sequence Detection System software (SDS version 2.2, PE Applied Biosystems, Foster City, CA, USA). Reaction mixtures contained 10 pmol/μl of each primer and 2X SYBR Green PCR Master Mix (PE Applied Biosystems) which contained HotStar Taq DNA-Polymerase in an optimized buffer, dNTP mix (with dUTP additive), SYBRs Green I fluorescent dye, and ROX dye as a passive reference. Each of the well had serial dilutions (1, 1/2 and 1/4, 1/8, 1/16) of cDNA to generate relative standard curves for genes. All primers (Table 3) were amplified using the same conditions. Thermal cycling conditions were as follows: 50 °C for 2 min, 95 °C for 10 min, followed by 40 cycles of 95 °C for 30 s, 60 °C for 30 s, 72 °C for 30 s. In order to exclude the presence of unspecific products, a melting curve analysis of products was performed routinely after amplification by a high-resolution data collection during an incremental temperature increase from 60 to 95 °C with a ramp rate of 0.21 °C/s.

### 4.9. Flow Cytometry

For surface staining, lymph node cells were incubated for 30 min at 4 °C with phycoerythrin (PE) anti-mouse TLR4 and allophycocyanin (APC) anti-mouse CD14 (eBioscience, Inc., San Diego, CA, USA). Isotype control antibodies were used to estimate the non-specific binding of target primary antibodies. These cells were analyzed using a flow cytometer (FACSAria III; Becton Dickinson, San Jose, CA, USA). For intracellular staining, cells were perforated with 0.5% saponin for 15 min at room temperature, than incubated with antibody for 30 min at 4 °C. Applied antidodies were anti-mouse PDCD4 (Bioss, Atlanta, GA, USA), RhoB (Bioss, USA), PD-1 (eBioscience, Inc., San Diego, CA, USA), and IL-12p35 (eBioscience, Inc., USA).

### 4.10. Measurement of Cytokines by ELISA

The serum levels of IL-17 and IL-6 were measured by ELISA (R&D Systems, Minneapolis, MN, USA). ELISA was conducted according to the manufacturer’s instructions. Results were read using a Bio-Rad (Hercules, CA, USA) model 170–6850 microplate reader at a wavelength of 450 nm. ELISA application was repeated three times in duplicate well.

### 4.11. Statistical Analysis

All data were expressed as mean ± S.D. Statistical differences between the experimental groups were determined using Student’s *t* test and Bonferroni correction. Statistical analysis was conducted using MedCalc^®^ version 9.3.0.0. (MedCalc Software bvba, Ostend, Belgium) Statistical significance was considered when *p* value was less than 0.05.

## 5. Conclusions

We confirmed that the level of miR-21 was correlated with BD in mice model and BD patients. miR-21 inhibition brought an improvement of HSV-induced BD-like inflammatory symptoms and down-regulated pro-inflammatory cytokine IL-17 and IL-6. A conventional medication to BD mice resulted in improvement accompanied with down-regulation of miR-21. miR-21 inhibition up-regulated PDCD4, PD-1, RhoB, and IL-12p35. miR-21 inhibition also resulted in down-regulation of the frequencies of TLR4+ cells. These findings suggest that the expression level of miR-21 is correlated with BD-like symptoms in a HSV induced mouse model. Therefore, miR-21 antagomir could be useful to control BD, ulceration caused by HSV infection, and other systemic inflammatory diseases as immune modulators.
